# Gaucher disease type 3 from infancy through adulthood: a conceptual model of signs, symptoms, and impacts associated with ataxia and cognitive impairment

**DOI:** 10.1186/s13023-025-03654-y

**Published:** 2025-04-10

**Authors:** Raphael Schiffmann, James Turnbull, Robert Krupnick, Ruth Pulikottil-Jacob, Chad Gwaltney, Alaa Hamed, Isabela Batsu, Walter Heine, Eugen Mengel

**Affiliations:** 1 Texas Christian University, Fort Worth, TX USA; 2 IQVIA Inc., New York, NY USA; 3 IQVIA Inc., Boston, MA USA; 4 Sanofi, Reading, UK; 5 Gwaltney Consulting, Westerly, RI USA; 6 Sanofi, Cambridge, MA USA; 7 Sanofi, Bridgewater, NJ USA; 8 SphinCS GmbH, Institute of Clinical Science in LSD, Hochheim, Germany

**Keywords:** Gaucher disease, GD3, Ataxia, Cognitive impairment, Conceptual model

## Abstract

**Background:**

Gaucher disease type 3 (GD3) is a lysosomal storage disease characterized by diverse neurological and systemic manifestations. Symptoms of ataxia, cognitive impairment, and other systemic symptoms profoundly impact daily activities and the quality of life for individuals living with the disease. Development of a conceptual model of disease for persons living with GD3 from birth to adulthood would enable objective monitoring of disease progression and assessment of treatment benefits.

**Methods:**

A targeted literature review, interviews with clinical experts, and interviews with individuals and their caregivers living in the UK and the US were carried out to understand the patient experience. Interviews were transcribed and de-identified data were analyzed to identify signs, symptoms, and impacts of ataxia, cognitive impairment, and other systemic impairments. A conceptual model was developed by integrating relevant signs, symptoms, and impacts experienced from birth through adulthood.

**Results:**

Review of symptoms and impacts of GD3 from three published scientific articles, and interviews with six clinical experts, 12 individuals living with GD3, and 12 caregivers, identified 58 patient experience concepts associated with GD3. Signs and symptoms associated with ataxia appear during the first 3 years of life and persist beyond 5 years of age, while signs and symptoms related to neurocognition appear later in life. Difficulty in shifting gaze and/or tracking objects, ataxia, tremors, memory problems, difficulty in processing new information, fatigue, and bone pain are most salient concepts for GD3. In patients aged ≤ 5 years, motor manifestations and symptoms were far more prevalent than neurocognitive signs and symptoms. Inability to work or perform at school, limited social and family engagements, restricted mobility (walking, driving, public transportation), and declining independence were the most important impacts on individuals with GD3.

**Conclusions:**

Heterogeneity exists in GD3 manifestations, especially neuromuscular and neurocognitive signs, symptoms, and impacts, across all age ranges of individuals living with GD3. The conceptual model developed in the study provided a comprehensive understanding of the disease in individuals with GD3.

**Supplementary Information:**

The online version contains supplementary material available at 10.1186/s13023-025-03654-y.

## Background

Gaucher disease (GD) is a rare lysosomal storage disease caused by deficient enzymatic activity of β-glucocerebrosidase, leading to accumulation of glucocerebroside in the lysosomes of monocytes and macrophages in liver, bone marrow, and spleen tissues [1]. The global prevalence and incidence of GD in the general population is 0.7–1.75 per 100,000 and 0.39–5.8 per 100,000, respectively [2]. Clinically, GD is characterized by hepatosplenomegaly, cytopenia, bone manifestations, and neurological impairment. The clinical phenotype of GD can be classified based on absence (type 1, chronic, non-neuronopathic) or presence of neurological deficits (type 2, acute, neuronopathic and type 3, chronic neuronopathic) [3]. GD type 2 manifests symptoms within the first 3–6 months of life and is typically fatal before 2 years of age. GD type 3 (GD3) has a more gradual onset compared with type 2, and patients with GD3 may survive into adulthood. The global prevalence of neuronopathic GD was found to be <1 in 100,000 live births [4]. Indeed, the neuronopathic form of GD including GD3 is predominant in northern Europe, Egypt, Japan, China, Korea, Taiwan, India, Africa, and Sweden; however, precise data are unavailable [5, 6]. GD3 presents with a wide variety of signs and symptoms. All patients with GD3 show signs of horizontal supranuclear gaze palsy [7], which remains isolated or accompanied by additional neurological and systemic symptoms, with overlapping clinical presentations across different life stages. Some patients exhibit slow horizontal saccadic eye movements, while others show slow progressive motor abnormalities, ataxia, spasticity, tremors, myoclonic seizures, organomegaly, developmental delays, and cognitive deficits [6, 8, 9]. Debilitating symptoms of the disease significantly affect activities of daily living (ADL). Hematologic and skeletal complications and fatigue can cause functional disability and adversely affect quality of life [10–12].

There is no cure for GD3, and once diagnosed, patients are usually treated with intravenous enzyme-replacement therapy (ERT), such as imiglucerase [13]. Individuals living with GD3 treated with ERT have shown improvement in visceral and hematological parameters and bone manifestations. However, these treatments have not yielded significant benefits in neurological manifestations of GD3 [12–14]. Newer investigational agents are being studied in clinical trials to determine whether they lead to improvements in neurological symptoms, including those related to ataxia and cognitive function [15]. As a result of the significant variation in the severity and onset of these neurological symptoms, and their impact on a range of daily activities [8], an in-depth understanding of signs and symptoms associated with ataxia and cognitive impairment will enable better understanding of the disease that can then be used to provide optimal care for patients [8]. The need for such understanding is particularly acute for pediatric patients, with knowledge about their experiences, onset of symptoms, and impact on their daily lives being limited [6, 8].

The primary goal of the present study was to identify signs, symptoms, and impacts associated with cerebellar ataxia, neurocognitive impairment, and systemic manifestations experienced by individuals living with GD3 from birth through adulthood. The study further aimed to develop a patient-centered conceptual model that includes neuromuscular, neurocognitive, and systemic signs, symptoms, and impacts for these individuals living with GD3.

## Methods

### Literature review

A targeted literature search was originally conducted to identify articles about adults living with GD3 (aged ≥ 18 years). PubMed, PsycINFO, and OVID databases (2004–2019) were searched to identify signs, symptoms, and immediate and general impacts of GD3 described in the medical literature. Data related to patient populations, endpoints, and findings describing the symptoms and impacts of GD3 were extracted. Three journal publications [16–18] and two published documents (Gaucher Registry Neurological Outcomes—First Assessment version Dec 16, 2010, Gaucher Disease Association and EGA Burden of Disease, June 10, 2014) were also reviewed.

An additional literature review to identify articles about the pediatric population (aged 0–18 years) living with GD3 was carried out using Google Scholar and PubMed®. The searches sought to identify reviews, observational studies, and non-interventional studies that were published within the last 15 years and were concerned with disease- or treatment-related symptoms and/or impacts experienced by children living with GD3. The search retrieved 152 relevant titles, of which three publications were selected after reviewing abstracts and full texts. Additionally, Google Scholar was searched for blogs and forums to understand the symptoms and experiences of individuals living with GD3. The search terms such as “Gaucher disease blog”, “Gaucher disease patients”, “Gaucher disease experience”, “My experience with Gaucher disease”, “Gaucher disease forum”, and “Gaucher disease patient forum” were used. The following patient blogs and forums were searched to identify first-person reports of patient experience: (1) Gauchers Association (https://www.gaucher.org.uk/); (2) Children’s Gaucher Research Fund (https://www.childrensgaucher.org/family-stories/); and (3) Patients and Families Living With Gaucher Disease (https://www.avrobio.com/patients-families/understanding-gaucher/living-with-gaucher-disease-gd3). Information from three articles identified in the literature review [9, 16, 19], six blogs about persons living with GD3, and 11 patients’ stories from patient and caregiver interviews were used for developing the preliminary conceptual model.

### Clinical expert interviews

Data on signs and symptoms of GD3 extracted from the published literature were first validated by a neuropsychologist for their alignment with developmental milestones. Interviews then were conducted with five expert clinicians to understand their perspectives on the signs, symptoms, and impacts of GD3. The three experts who participated in the first round of clinical interviews were a consultant hematologist and two metabolic medicine specialists from the United Kingdom (UK), Poland, and Japan, respectively. In the second round, two principal investigators, one from Germany and the other from the United States (US), were interviewed to determine observed changes in the GD3 experience as part of the ongoing LEAP clinical trial (NCT02843035). They were recruited from a list of GD3 clinician experts who managed individuals living with GD3 at metabolic referral centers. Upon invitation, each clinician participated in a 40- to 60-min telephone interview. A structured interview guide was used to elicit insights on the patient experiences, signs, and symptoms of GD3, and the impact of disease on patients’ daily activities across pediatric, adolescent, and adult age groups. During the interviews, clinical experts were presented with a preliminary GD3 conceptual model and suggestions for further modifications were solicited.

### Interviews of patients living with GD3 and their caregivers

Patients living with GD3 who were aged 17–59 years and were from the UK and the US were interviewed between February 2016 and May 2019 to collect information about their symptoms over their lifespan, especially regarding their experiences with ataxia and cognitive impairment. In the UK, Gauchers Association facilitated identification of patients who were willing to participate in a 60- to 75-min interview. In the US, a research coordinator at a LEAP clinical trial site facilitated identification of patients willing to participate. IQVIA shared the study details with the patients who confirmed their participation and provided informed consent. Patients were eligible if they had a GD3 diagnosis, were enrolled in the LEAP trial (US only), were willing and able to provide informed consent (or their parent or legally authorized representative was able to do so) and participate in the interview, and had no history of neurologic events or mental conditions that would prevent them from understanding the nature, scope, and possible consequences of the study. During their interviews, patients were probed to describe their signs and symptoms, the impacts of GD3, when the signs and symptoms began, and how these experiences changed over time.

Caregivers were considered eligible if they were a primary caregiver for a patient with GD3, were willing and able to provide informed consent and participate in the interview, and had no history of neurologic events or mental condition that would prevent them from understanding the nature, scope, and possible consequences of the study. Gauchers Association (UK) supported identification of caregivers willing to participate in a 75-min telephone interview. Caregivers of patients enrolled in LEAP trial in the US were eligible to participate in the interview. In the US, a research coordinator at a LEAP clinical trial site facilitated identification of caregivers willing to participate. IQVIA shared the study details with the caregivers who confirmed their participation and provided informed consent. During their interviews, UK caregivers described their experiences with care recipients over their lifespan, and US caregivers discussed changes experienced prior to enrollment in the LEAP trial.

During interviews, both patients and caregivers were probed about the earliest signs/symptoms, their frequency, timing, and severity of symptom occurrence, and how these symptoms progressed in terms of severity. Patients were able to provide a time period during which symptoms first appeared, rather than pointing to a specific age at which symptoms became apparent. Patients and caregivers could recall experiences with GD3 during childhood (<12 years), adolescence (12–17 years), and adulthood (>18 years), followed by the impacts these symptoms had on patients during that phase of their life. The Advarra Institutional Review Board (IRB) approved the study (on November 29, 2018) and standardized interview guides, which were used to conduct patient interviews in the UK and US. Interviews were conducted as per the recommendations in guidelines from the International Society of Pharmacoeconomics and Outcomes Research Good Research Practices Task Force.

### Data analysis

Interviews were recorded and audio files were transcribed for data analysis. De-identified transcripts of interviews of the clinical experts, patients, and caregivers were analyzed, and relevant data were extracted. Data from interviews were grouped into a library of signs, symptoms, and impacts to allow quantification and to systematically uncover relevant concepts. After coding was completed, the frequency of concepts mentioned and the proportion of spontaneous to probed mentions were generated to determine the salience of concepts, along with patient and caregiver ratings of the “bothersomeness” or “disturbance” of these concepts.

## Results

The age of patients (n = 12) at the time of the interviews ranged between 17 and 59 years. Eight of the 12 patients interviewed were female, and nine of them lived in the UK (Table 1). Ten were diagnosed with GD3 before the age of 2 years, and after diagnosis, all patients received ERT every 2 weeks. At the time of the interviews, three patients were receiving investigational therapy (venglustat in combination with imiglucerase) in the LEAP trial. The age range of caregivers (n = 12) at the time of the interviews was between 36 and 58 years old. Eleven of the 12 caregivers interviewed were a parent of a patient with GD3, nine were aged >40 years, and eight were in the UK (Table 2).

**Table 1:** Demographics of patients living with GD3 included in the study

Patient gender	Age (in years) at interview	Age at diagnosis	Region	Treatment
Female	17	18 months	UK	ERT Q2W since diagnosis
Female	19	18 months	UK	ERT Q2W since diagnosis
Male	20	18 months	US	Spinal-cord fusion surgery at age 15; ERT Q2W since diagnosis; now on experimental LEAP drug
Female	21	16 months	UK	ERT Q2W since diagnosis
Female	22	15 months	UK	ERT Q2W since diagnosis
Female	22	Birth	UK	ERT Q2W since age 11 years; now on velaglucerase alfa for 2 years
Male	24	18 months	UK	ERT Q2W since diagnosis
Female	25	23 months	US	Hip-replacement surgery at age 24; ERT Q2W since diagnosis; now on experimental LEAP drug
Male	27	15 months	US	ERT Q2W since diagnosis; now on experimental LEAP drug
Female	29	9 years	UK	ERT Q2W since age 11 years; now on velaglucerase alfa
Female	54	Birth	UK	Splenectomy (at age 5 years); ERT Q2W since diagnosis; teriparatide for bones
Male	59	5 years	UK	ERT Q2W since age 18 years; now on velaglucerase alfa for 1 year

*ERT* enzyme-replacement therapy, *GD3* Gaucher disease type 3, *Q2W* every 2 weeks, *UK* United Kingdom, *US* United States

**Table 2:** Demographics of caregivers for patients living with GD3 included in the study

Caregiver gender	Caregiver role	Caregiver age (years)	Care-recipient gender	Care-recipient age (in years)	Care-recipient age at diagnosis	Region
Female	Grandmother	42	Male	4	24 months	UK
Female	Mother	37	Male	7	18 months	UK
Female	Mother	36	Female	7	18 months	UK
Male	Father	58	Female	20	18 months	UK
Female	Mother	51	Male	20	16 months	US
Male	Father	51	Female	25	18 months	UK
Female	Mother	57	Female	25	Birth	UK
Female	Mother	49	Female	25	17 months	UK
Female	Mother	–	Female	25	13 months	UK
Male	Father	48	Female	25	23 months	US
Female	Mother	50	Male	27	15 months	US
Female	Mother	56	Male	28	24 months	US

*GD3* Gaucher disease type 3, *UK* United Kingdom, *US* United States

### Clinical manifestations and impacts associated with ataxia

Data collected from literature reviews, patient blogs, and qualitative interviews indicated that signs and symptoms associated with ataxia are common in children aged ≤ 5 years who are living with GD3. These symptoms continue to affect children living with GD3 through adolescence and into adulthood. Additional file 1: Table S1 presents common concepts associated with ataxia and indicates the ages at which symptoms appear between infancy and adulthood. The early symptoms experienced by pediatric patients within the first 12 months of their birth include walking problems and trouble with balance along with signs of deep tendon reflexes. In the first 3 years from birth, toddlers living with GD3 experienced additional signs and symptoms such as abnormal fine motor skills, retroflexion of the head/spinal alignment, swallowing difficulties, spasticity, limited gross motor skills, and muscle weakness. Pre-school children (up to 5 years of age) living with GD3 further experienced frequent falls, chewing difficulties, and speech problems in addition to existing ataxia-associated symptoms. Lack of coordination became apparent in children living with GD3 by 6 years of age. Children continued to suffer from these symptoms throughout their adulthood.

Adult patients (92%, 11/12) and caregivers (92%, 11/12) spontaneously mentioned eye-movement abnormalities that were present from birth through adulthood (Table 3). Issues with horizontal movement of eyes from left to right and vice versa, an inability to voluntarily shift gaze, difficulty in tracking moving objects, and needing to move their whole head to see were the most reported eye-movement problems experienced by patients living with GD3. A caregiver from the UK described her care-recipient’s difficulty with eye movement as follows:

She always finds it very difficult if there’s a room full of people and someone is calling her from one side and then someone is calling her from the other side, because, like I said, when she turns her head, she has to close her eyes and turn her head. She can’t just roll her eyes from one side to the other to see who’s calling her.

**Table 3:** GD3 signs and symptoms in patients aged ≥18 years reported by patients and caregivers

Patient signs and symptoms	Sign or symptom	Domain	Frequency of sign or symptom mentioned		Spontaneous/probed mentions	
			Patient n (%)	Caregiver n (%)	Patient	Caregiver
Eye-movement issues	Symptom	Ataxia-associated	11 (92)	11 (92)	9/2	9/2
Fatigue (feeling tired/having low energy)	Symptom	Systemic	10 (83)	8 (67)	8/2	4/4
Enlargement of the liver and spleen	Sign	Visceral	6 (50)	11 (92)	6/0	11/0
Difficulty understanding new information quickly/slow to process new information	Symptom	Neurocognitive	6 (50)	10 (83)	1/5	7/3
Lack of balance	Symptom	Ataxia-associated	8 (67)	6 (50)	5/3	6/0
Bone pain	Symptom	Systemic	7 (58)	6 (50)	5/2	3/3
Memory loss/trouble with memory	Symptom	Neurocognitive	4 (33)	6 (50)	1/3	4/2
Curvature of the spine	Symptom	Systemic	5 (42)	5 (42)	5/0	3/2
Uncontrollable movements (tics and/or tremors)	Symptom	Ataxia-associated	5 (42)	4 (33)	5/0	2/2
Difficulty solving problems/making decisions	Symptom	Neurocognitive	3 (25)	6 (50)	0/3	0/6
Vision problems	Symptom	Systemic	5 (42)	3 (25)	5/0	3/0
Breathing difficulties	Symptom	Systemic	4 (33)	4 (33)	1/3	4/0

*GD3* Gaucher disease type 3

Some key impacts experienced by adult patients due to impairment of eye movement included driving a car by themselves, going out in public (e.g., crossing the road, using public transportation, going into crowds), playing sports, reading unassisted, ascending or descending stairs or escalators, and having conversations with multiple people (e.g., shifting between conversations).

Impaired balance was a commonly observed manifestation of ataxia among patients (67% [8/12]) and caregivers (50% [6/12]). They described impaired balance as having weak or poor balance or lack of movement coordination (Table 3). An example reported by a UK caregiver is as follows:

She doesn’t have balance. She gets toppled over easily. Crowds and unfamiliar places, busy places, are triggers to make it a lot worse.

Issues with balance and walking caused children to frequently bump into household objects such as tables and sofas. To avoid bumps and falls, children living with GD3 would avoid taking stairs, or going into crowded places and keep away from physical exercises. They also could not play or run outside for long, as they would tire quickly. Consequently, reduced interactions with other children on playgrounds affected their school friendships as they were unable to make many friends. One caregiver from the UK described the impact that difficulties with her balance and walking had on the patient’s daily activities.

Well, her balance and her normal walking wasn’t right. She was bumping into objects, tables, into sofas. This is a daily thing. When she was younger, actually, she wasn’t walking properly a straight line. As time progressed…actually, progressed to her leg as well. It got worse.

Impaired balance also affected adult patients who faced difficulty with walking in crowds, going out by themselves, ascending or descending stairs, and exercising.

Uncontrollable movements (tics and/or tremors) were reported as a symptom of GD3 by 42% (5/12) of patients and 33% (4/12) of caregivers (Table 3). They were described as tremors and shakes, experienced by patients every day, at any time, mostly in their hands. The severity of the tremor and the onset of tremors appeared to vary. Patients and caregivers reported tremors beginning in childhood, whereas multiple caregivers reported the symptom first occurring at 17 or 18 years of age. Tremors in children impacted their ability to write in school or hold a cup and walk with it. Adults with tremors were unable to perform certain household activities (e.g., holding a cup, cutting food, fastening buttons). One patient from the UK reported avoiding eating in public because of her tremor.

Very noticeable in the kitchen. I can’t carry hot drinks; I spill a lot. I had to give up self-cannulation. Doing up buttons is also difficult. Anything that requires fine motor skills, including writing, is difficult. Left hand is worse. Every day. I’m seeing a neurologist about this. I tried beta blockers, but they didn’t work. Got worse.

These impacts of clinical manifestations of ataxia were observed across the age spectrum of patients living with GD3, with specific circumstances illustrated based on the participant's age (e.g., school-related activities). Ataxia was noted as impacting the quality of life in some aspects across all ages.

### Clinical manifestations of cognitive impairment

Results from literature reviews and qualitative interviews suggested that cognitive impairment is not readily apparent in infants and newborns living with GD3. These signs and symptoms first appear between 3 and 6 years after birth and continue throughout adulthood. Additional file 1: Table S2 displays concepts associated with cognitive impairment in patients living with GD3 across different stages of life. Symptoms of cognitive impairment or regression appeared within the first 3 years after birth, and by 5 years of age (pre-school children), impulsive behavior, delays with speech, and developmental delay/regression were noticeable. In children living with GD3, explosive speech, diminished intelligence, dementia or problems with memory, trouble organizing thoughts, difficulty in solving problems or making decisions, and difficulties with processing new information became apparent by 6 years of age. Children continued to experience these symptoms throughout adulthood.

According to the clinician experts interviewed in the study, about 30%–67% of adult patients living with GD3 in general are too cognitively impaired to self-report their experiences of the disease. During patient and caregiver interviews in this study, 50% (6/12) of patients and 83% (10/12) of caregivers reported difficulty in understanding new information quickly as one of the symptoms of GD3 (Table 3). Caregivers were more likely than patients to spontaneously mention this symptom. Patients were slow at processing verbal information, experienced difficulties in understanding new information, or experienced an auditory delay. These symptoms had been present since childhood and were more pronounced in school, where patients had to deal with verbally transferred information daily. A UK caregiver described her care-recipient’s cognitive difficulties during school as follows:

When she was in secondary school, she found it very difficult to process the information at school. She needed more time during her exams, she couldn’t really focus on the work. She found education quite difficult.

One-third of interviewed patients (33%, 4/12) and 50% of caregivers (6/12) mentioned trouble with memory, which typically worsened with increasing age (Table 3). Patients and caregivers described memory issues as being forgetful, having difficulty in retaining information, or having poor short-term memory. Many caregivers spontaneously mentioned memory issues as a symptom; however, patients mentioned memory problems mostly when probed about this experience. Caregivers reported that memory issues became more noticeable during adolescence and adulthood. Difficulties with memory and their impact on daily life were described by a UK patient.

I do have memory trouble. It’s nothing alarming; I remember things from a long time ago but won’t remember things that I have done recently. Someone will say can you go downstairs and get me X and I’ll forget what I came for. ‘What am I doing in this room?’ It makes me panic, I’ve been in a position when I lost my way home. I became hysterical. I’m supposed to know that route. Weekly basis. Not daily.

This patient’s caregiver reported her experience with the patient’s cognitive impairment as follows:

It’s getting worse. She can be very forgetful. I think it’s always been there, but I didn’t notice it too much because I was doing too much for her. As she became more independent, you could say that she would forget appointments. She would put things in places and forget where she put them. She doesn’t forget events.

Difficulty in solving problems/making decisions was reported by three patients (25%, 3/12) and six caregivers (50%, 6/12; Table 3). Difficulty in solving problems/making decisions was reported as a daily, constant symptom that has always been present in the patients’ lives without changing much in severity with increasing age. Patients needed constant help in figuring out basic tasks and were at risk of making irrational/unreasoned decisions. This caused patients to become frustrated, and emotionally upset, as shown in the following quote from a UK caregiver.

He has trouble making reasoned decisions. You cannot really reason with him. So, he can decide not to do something or to do something, but you can't explain to him why that's not a good idea without there being a protracted problem, if that makes any sense.

Both pediatric and adult patients experienced impacts on their lives due to cognitive impairment. Difficulty in understanding or processing new information caused repetitions during verbal conversations, often leading to embarrassment to the patient. Patients also needed assistance in taking notes, which affected their performance in school as they had to repeat subjects or needed longer time for examinations. Memory issues impacted the patient’s ability to learn in school and caused difficulty in performing tasks integral to adolescence or adult life (e.g., planning/remembering appointments, finding their way home). Patients who faced difficulties in making decisions needed assistance to complete forms or to make financial decisions.

### Other clinical manifestations

Literature reviews, patient forums, and qualitative interviews indicated that splenomegaly, hepatomegaly, and thrombocytopenia were common systemic signs in children living with GD3 (aged ≤ 5 years). Anemia, growth retardation, vertebral body collapse, bone pain, bone crises, and enlarged abdomen were other systemic signs or symptoms experienced by patients of this age group (Additional file 1: Table S3). Clinical experts observed that children living with GD3 appeared to experience bone pain, fatigue, and seizures at diagnosis (age range at diagnosis, 2–5 years). Clinical experts believed ERT provided a strong clinical benefit, although individuals living with GD3 continued to suffer from a range of symptoms during their lifetime.

In qualitative interviews, these additional clinical symptoms were also mentioned by patients and caregivers, with most adult patients (83%, 10/12) and caregivers (67%, 8/12) described fatigue as an additional key symptom of GD3 (Table 3). Patients spontaneously mentioned fatigue more often than caregivers. Patients experienced fatigue as getting tired easily, feeling tired, having low energy, and being down. All patients and caregivers noted an age-dependent increase in the severity and frequency of fatigue; most patients linked fatigue to ERT infusions received every 2 weeks. Some patients noted that continuously compensating for eye-movement issues increased the levels of fatigue and affected their ability to have a good night’s sleep. Other factors such as daily activities/responsibilities (e.g., work, school, sports) also impacted the degree of fatigue. At work/school, patients experienced fatigue due to increased stress and trouble in concentrating and were more prone to getting flustered/emotional, which affected the patients’ overall performance. An example of this fatigue is described in the following quote from a UK patient:

More frequent towards the end of ERT. ERT gives me a little boost which only lasts a few days and then I get gradually more tired as the fortnight goes on. If I have a lot of activities or have a lot on or must concentrate on things to get done, I get more stressed which results in more fatigue. The weather can also affect me. If I’m too warm or feel I’m getting flustered. I get flustered very easily and emotional; the room spins and that makes me more tired.

Some patients (42%, 5/12) and caregivers (25%, 3/12) spontaneously reported a variety of vision problems (Table 3). Commonly reported vision problems, such as lack of peripheral vision, (partial) blindness, glaucoma, blurry vision, needing a magnifying glass to read, and having a “lazy” eye, were distinct from eye-movement abnormalities listed earlier, under clinical manifestations associated with ataxia. Patients often bumped into objeccts and had difficulty in reading. Vision problems seemed to limit patients’ independence, as mentioned in the following quote.

Can’t really see through my right eye. It’s quite blurry compared to my left. I have a tube fitted to help with pressure. Doesn’t matter what surgery I have, it feels like it will be complicated by my condition. I have had seven operations so far. I wake up during the night because of eyes. Blurry vision. My vision can be double. Really tires me out during the day. Eye drops make me tired and give me headaches.

Bone pain was reported by 58% (7/12) of patients and 50% (6/12) of caregivers as another common manifestation of the disease (Table 3). Patients described their experience as deep pain, ache, and stiffness in the back and extremities. Bone pain was reported to have started during adolescence and adulthood. Individuals living with GD3 experiencing bone pain often experienced additional pain due to surgeries such as hip replacement and spinal fusion. The following quote describes bone pain in one patient.

The bones ache a lot, which don’t make me feel my age; it makes me feel somewhat old. Deep pain. It stops me from doing basic activities and lifting things and moving around. We’ve recently had an extension done for a downstairs bathroom because going up and down stairs can be a struggle. Washing and dressing is also difficult. I couldn’t run for more than about 10 mins and used to be told off in physical education because the teachers didn’t know what GD3 was. It was the same in secondary school.

As per clinician experts interviewed in the study, lack of confidence or self-esteem and worrying about sustaining a relationship or getting married were common impacts of systemic symptoms. Some adult and pediatric patients living with GD3 experienced distress, while some adult patients experienced feelings of depression, followed by apathy and difficulties in coping. Patients also feared missing infusions as ERT slows or stops systemic progression of GD3. Symptoms of fatigue prevented both adult and pediatric patients from participating in social activities. Daily activities that were impacted by bone pain in adult patients included lifting objects, moving about, climbing stairs, washing, and dressing. Bone pain also interfered with proper relaxation, finding a comfortable position to sit in or sleep at night. Adult patients further experienced reduced ability to work and an impaired ability to carry out family obligations or manage finances.

### GD3 conceptual model

**Fig. 1:**
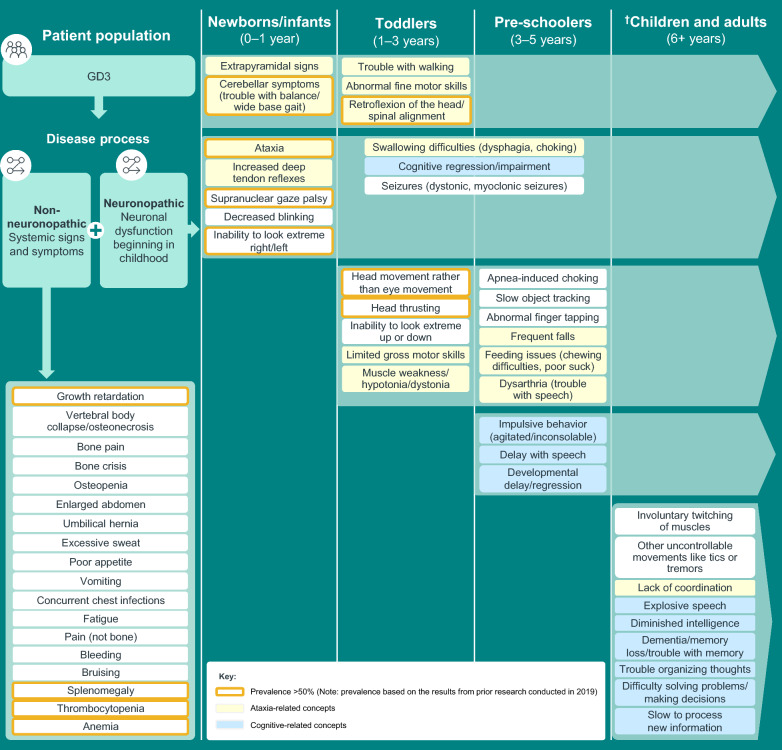
Model for typical onset of GD3 concepts associated with ataxia, cognitive impairment, and systemic signs and symptoms. Symptoms are listed during the periods in which they were first reported and continue during the remainder of the lifespan (indicated by the lighter green arrow). ^†^Includes results from prior research conducted in 2019 and documented in the report, “Understanding and Measuring the Patient Experience with Gaucher Disease Type 3.” *GD3* Gaucher disease type 3

A conceptual model of GD3 was developed from the integration of key concepts identified from the literature reviews, clinical expert interviews, and patient and caregiver interviews. The conceptual model based on our findings shows the most prominent neuromuscular and neurocognitive signs and symptoms of GD3 and the stage(s) of life at which their onset occurs (Fig. 1). Signs and symptoms associated with ataxia appear during the first 3 years of life and persist beyond 5 years of age (Additional file 1: Fig. S1 and Table S4). Signs and symptoms related to neurocognition appear later in life (Additional file 1: Fig. S2 and Table S4). Growth retardation, splenomegaly, thrombocytopenia, bone pain, anemia, and fatigue are common visceral or systemic symptoms of individuals living with GD3 (Fig. 1). The most commonly reported impacts of GD3 included a reduced ability to perform at school/work, declining independence (only patients aged >18 years), limitations in social/familial engagements and leisure activities, and frequent medical appointments.

## Discussion

The present study developed a conceptual model for understanding the experiences of patients living with GD3 from birth through adulthood. The study further investigated patient experiences of signs and symptoms associated with ataxia and cognitive impairment, and identified the impact these symptoms had on daily activities across all stages of life from birth to adulthood. Concepts from literature reviews, clinician interviews, and interviews with patients and caregivers identified 58 concepts associated with GD3. Clinical manifestations of GD3, including neurologic manifestations, were heterogeneous, and several of these manifestations affected daily activities of patients across all life stages. The present study further identified domains common to both pediatric and adult patients, such as ataxia and cognitive impairment; however, the severity and manifestation of these symptoms within these domains varied among patients. This study also showed that the perspectives of caregivers about symptoms of GD3 mostly aligned with experiences described by patients.

Published data suggest ataxia is experienced as a neurological symptom by about 15%–50% of individuals living with GD3 [3, 9, 16]. However, in this study, the burden of symptoms associated with ataxia was much higher among patients (eye-movement issues; 92%; impaired balance, 62%; and uncontrollable movements, 42%). It is important to note that “ataxia” in this study is used as a collective term for a range of symptoms, including strabismus, balance problems, and coordination difficulties, among others. However, earlier studies often referred to “ataxia” as a singular concept or neurological sign. Hence, the burden of ataxia might not be greater than what was previously reported, highlighting the differences in the definition of the term across various studies.

Capturing the changes in the symptoms associated with ataxia in patients living with GD3, especially in clinical studies, appears to be feasible with use of the Scale for Assessment and Rating of Ataxia (SARA) [15, 20, 21]. SARA is a scale designed for use in clinical trials to measure a range of impairments due to cerebellar ataxia [22]. In validation studies carried out in patients living with spinocerebellar ataxia, SARA showed good construct validity, inter-rater validity, and sensitivity to change [22, 23]. Further, the SARA score for ataxia showed a good correlation with the Barthel ADL index and the Unified Huntington’s Disease Rating Scale (UHDRS®) functional assessment [22, 24]. This suggests that the eight items measured by the SARA tool (gait, stance, sitting, speech, finger-chase test, nose-finger test, fast alternating movements, and heel-shin test) were related to a patient’s ability to carry out ADL. For example, the severity of lack of coordination in patients living with GD3 can be captured by the quantitative scores from each of the SARA tool categories listed above. The severity of problems with walking, balance, and ataxia in patients can be measured by the gait, stance, and sitting items of SARA. Similarly, severity of strabismus in patients can be captured by the finger-chase item of the SARA scale. A shift in the SARA score due to disease progression or treatment may be reflective of changes in the severity of symptoms associated with ataxia in patients and might be associated with change in their ability to perform ADL.

Individuals living with GD3 often experience developmental delays, learning disabilities, cognitive deficits, and dementia [6]. Typically, 60% of individuals living with GD3 have below-average intellectual skills and visual-spatial dysfunction, and these symptoms are resistant to ERT [25]. Approximately 50%–83% of the caregivers in this study also described symptoms of cognitive impairment in adults living with GD3. Changes in the symptoms related to attention and immediate memory deficits in patients have been measured with the Repeatable Battery for Assessment of Neuropsychological Status (RBANS) [26, 27], and the same tool appears to be useful for evaluating cognitive improvements associated with experimental treatments. This scale can be used to measure cognitive deficits in patients living with GD3 in five cognitive domains—immediate memory, visuospatial and constructional function, language, attention, and delayed memory [28]. RBANS has been clinically validated to identify and characterize cognitive impairment across diverse neurodegenerative diseases [29]. Further, RBANS scores, particularly those in immediate memory, attention, and visuospatial/constructional domains, strongly correlate with measures of daily living [30]. The concepts related to cognitive impairment in the model, such as difficulty in understanding new information quickly, memory deficits, and issues with problem solving, may be captured by all five domains of RBANS and their severity can be scored. Hence, a change in RBANS score due to disease progression or treatment may reflect a change in the severity of cognitive dysfunction in patients. Thus, RBANS may be a potential tool for capturing changes in cognitive functions and may relate to measures of daily living in participants of GD3 clinical trials.

The study has several limitations. The concepts of GD3 symptoms elicited may be biased to include those associated with Gauchers Association (UK) and/or participants of the LEAP clinical trial. The study's exclusive focus on UK and US patients introduces a sample bias, and consequently, the findings may not be generalizable to individuals from other regions with distinct GD3 phenotypes (for example, Norrbotten in Sweden). Potential sample bias could arise from the patient selection criteria of the LEAP clinical trial, which excludes patients with a history of significant neurological events such as stroke or epilepsy. Clinical data on the genotype and enzyme activities of the patients at diagnosis were not collected and analyzed; therefore, heterogeneity of the manifestations of GD3 even within a relatively homogeneous group of affected individuals in this study could not be ascertained. Further, myoclonic and early liver failure phenotypes could not be captured efficiently in the UK and US population. In addition, while the interview guides and analysis approach allowed for a mapping of concepts to age intervals akin to developmental milestones, it is possible that the age thresholds used to categorize different milestone periods are imprecise. Furthermore, patients with limited access to internet and telephonic facilities and those not involved in LEAP clinical trial may not be represented. The small sample size of patients and caregivers interviewed may not be adequate to identify less prevalent concepts of GD3. Small sample size of patients and caregivers from the UK and the US may not be adequate to generalize GD3 disease concepts. Also, recall bias may be noteworthy, especially in patients experiencing cognitive symptoms. Finally, patients with mild symptoms may have been more willing to participate in 60- to 75-min interviews, while those with severe pain, fatigue, and cognitive dysfunction along with severely impacted quality of life may not have been motivated to take part in these interview.

## Conclusions

Heterogeneity exists in GD3 manifestations, especially neuromuscular and neurocognitive signs, symptoms, and impacts, across all age ranges of patients living with GD3. Caregivers typically share the patients’ perspectives on the experience of these symptoms. Signs and symptoms associated with ataxia and cognitive impairment, in particular, affected daily activities of the patients. Eye-movement abnormalities, lack of balance, and tremors are GD3 signs and symptoms that may be assessed by SARA, whereas an inability to understand new information quickly, memory problems, and difficulties in problem solving may be captured by RBANS. The conceptual model developed in the study supports the use of RBANS and SARA to measure GD3-relevant endpoints in ongoing clinical trials.

## Supplementary Information

Additional file 1.

## Data Availability

Data underlying the findings described in this article may be obtained in accordance with the Sanofi data-sharing policy described at https://www.sanofi.com/en/science-and-innovation/clinical-trials-and-results/our-data-sharing-commitments.

## Abbreviations

ADL: Activities of daily living
ERT: Enzyme-replacement therapy
GD: Gaucher disease
GD3: Gaucher disease type 3
IRB: Institutional Review Board
RBANS: Repeatable Battery for Assessment of Neuropsychological Status
SARA: Scale for Assessment and Rating of Ataxia
UK: United Kingdom
US: United States
VPRIV: Velaglucerase alfa for injection
