# Supplementary material for: Gaucher disease type 3 from infancy through adulthood: a conceptual model of signs, symptoms, and impacts associated with ataxia and cognitive impairment
Source: Orphanet J Rare Dis. 2025 Apr 10;20:171. doi: 10.1186/s13023-025-03654-y (PMC11987173; doi:10.1186/s13023-025-03654-y)
Supplement: Supplementary file 1 — Additional file 1. [file 13023_2025_3654_MOESM1_ESM.docx]

# Additional file 1

## Supplementary Tables and Figures

## Table S1 Key concepts associated with ataxia and their onset in patients living with GD3

| **Concepts related to ataxia** | **Onset of symptom (age)** | | | |
| --- | --- | --- | --- | --- |
|  | **Newborns/ infants  (0–1 year)** | **Toddlers  (1–3 years)** | **Pre-schoolers  3–5 years)** | **Children and adults  (≥6 years)** |
| Trouble with walking | + | + | — | — |
| Extrapyramidal signs | + | + | — | — |
| Cerebellar symptoms (trouble with balance/wide base gait) | + | + | — | — |
| Abnormal fine motor skills | — | + | — | — |
| Retroflexion of the head/spinal alignment | — | + | — | — |
| Ataxia | + | + | + | — |
| Increased deep tendon reflexes | + | + | + | — |
| Swallowing difficulties (dysphagia, choking) | — | + | + | — |
| Limited gross motor skills | — | + | + | — |
| Muscle weakness/hypotonia/dystonia | — | + | + | — |
| Frequent falls | — | — | + | — |
| Feeding issues (chewing difficulties, poor suck) | — | — | + | — |
| Dysarthria (trouble with speech) | — | — | + | — |
| Lack of coordination | — | — | + | + |

Note: The “+” sign indicates the symptom when it was first observed and, after onset, these symptoms persisted beyond 6 years of age in patients living with GD3.

GD3: Gaucher disease type 3

## Table S2 Key concepts associated with cognitive impairment and their onset in patients living with GD3

| **Concepts related to cognitive impairment** | **Onset of symptom (age)** | | | |
| --- | --- | --- | --- | --- |
|  | **Newborns/ infants  (0–1 year)** | **Toddlers  (1–3 years)** | **Pre-schoolers  3–5 years)** | **Children and adults  (≥6 years)** |
| Cognitive regression/impairment | — | + | + | — |
| Impulsive behavior (agitated/inconsolable) | — | — | + | — |
| Developmental delay/regression | — | — | + | — |
| Explosive speech | — | — | — | + |
| Diminished intelligence | — | — | — | + |
| Dementia/memory loss/trouble with memory | — | — | — | + |
| Trouble organizing thoughts | — | — | — | + |
| Difficulty solving problems/making decisions | — | — | — | + |
| Slow to process new information | — | — | — | + |

Note: The “+” sign indicates the symptom when it was first observed and after onset these symptoms persisted beyond 6 years age in patients living with GD3.

GD3: Gaucher disease type 3

## Table S3 Common GD3 systemic signs and symptoms in patients aged ≤5 years

| **Systemic signs and symptoms^a^** | **Highest prevalence (%)** | **Visual prevalence** | **Number of sources mentioning the concept** |
| --- | --- | --- | --- |
| Splenomegaly | 100 | >50% | 5 (a, c–e) |
| Hepatomegaly | 97 | >50% | 4 (a, c–e) |
| Thrombocytopenia | 70 | >50% | 2 (a, c) |
| Anemia | 60 | >50% | 2 (a, c) |
| Growth retardation | 56 | >50% | 1 (a) |
| Vertebral body collapse/osteonecrosis | 20 | 20–49% | 1 (c) |
| Bone pain | 11 | <20% | 2 (a, e) |
| Bone crisis | 10 | <20% | 2 (a, c) |
| Enlarged abdomen | N/A | N/A | 6 (d, e) |
| Umbilical hernia | N/A | N/A | 1 (d) |
| Excessive sweat | N/A | N/A | 1 (d) |
| Poor appetite | N/A | N/A | 1 (e) |
| Vomiting | N/A | N/A | 1 (e) |
| Concurrent chest infections | N/A | N/A | 2 (c, d) |

^a^Concepts were aggregated from all sources, which cover articles, patient forums, and qualitative interviews. Prevalence: percentage of patients mentioning a symptom.

Sources: (a) Tylki-Szymańska et al., 2010; (b) Davies et al., 2007; (c) Kraoua et al., 2011; (d) Patient forum; (e) Qualitative interviews.

GD3: Gaucher disease type 3; N/A: not applicable

## Table S4 Neuromuscular and neurocognitive signs and symptoms of GD3 in patients aged ≤5 years

| **Neurological sign or symptom** | **Highest prevalence of patients mentioning sign/symptom^1^** | **Age (in months) when signs or symptoms presented^2^** | **Number of sources mentioning sign or symptom** |
| --- | --- | --- | --- |
| Inability to look extreme right or left (horizontal gaze palsy) | 100% | 5–48 | 7 (a–e) |
| Ataxia | 80% | 5–36 | 3 (b–d) |
| Head movement rather than eye movement | 63% | 24–48 | 4 (a, d, e) |
| Head thrusting | 55% | 24 | 1 (a) |
| Cerebellar symptoms (trouble with balance/wide-based gait) | 53% | 5–36 | 4 (a–d) |
| Retroflexion of the head/spinal alignment | 53% | 3–20 | 2 (a, b) |
| Inability to look extreme up or down | 45% | 24 | 1 (a) |
| Seizures (dystonic, myoclonic seizures) | 44% | 9–17 | 4 (a–d) |
| Slow object tracking | 43% | 24 | 1 (a) |
| Swallowing difficulties (dysphagia, choking) | 40% | 3–48 | 6 (a–e) |
| Dysarthria (trouble/delay with speech) | 33% | 48 | 4 (a, b, e) |
| Limited gross motor skills | 32% | 17 | 1 (d) |
| Muscle weakness/dystonia | 25% | 13–17 | 2 (a, c) |
| Increased deep tendon reflexes | 23% | — | 1 (a) |
| Extrapyramidal signs | 21% | 3–36 | 2 (a, b) |
| Trouble with walking | 15% | 13–18 | 3 (a, c, d) |
| Cognitive regression/impairment | 13% | 5–60 | 3 (b, c, e) |
| Feeding issues (chewing difficulties, poor suck) | 11% | 17 | 1 (a) |
| Abnormal finger tapping | 11% | 60 | 1 (a) |
| Frequent falls | 10% | 16–48 | 3 (c–e) |
| Developmental milestones delay/regression | 10% | 48 | 1 (c) |
| Abnormal fine motor skills | 10% | 13 | 1 (a) |
| Impulsive behavior (agitated/inconsolable) | NR | 24 | 3 (d, e) |
| Apnea-induced choking | NR | 17 | 5 (a, e) |
| Decreased blinking | NR | — | 1 (d) |

^1^Concepts were aggregated from all sources (i.e., articles, blogs/forums, and qualitative interviews). Shown for each sign or symptom is the highest prevalence mentioned from the different sources.

^2^Age ranges were from all sources that mentioned the sign or symptom.

Sources: (a) Tylki-Szymańska et al., 2010 (cohort size, 131 patients); (b) Davies et al., 2007 (cohort size, 55 patients); (c) Kraoua et al., 2011 (cohort size, 10 patients); (d) Patient forum(s) (cohort size, 1 patient each); (e) Qualitative interviews.

GD3: Gaucher disease type 3; NR: not reported (in any source)

## Fig. S1 Conceptual model for onset of GD3 signs and symptoms associated with ataxia*


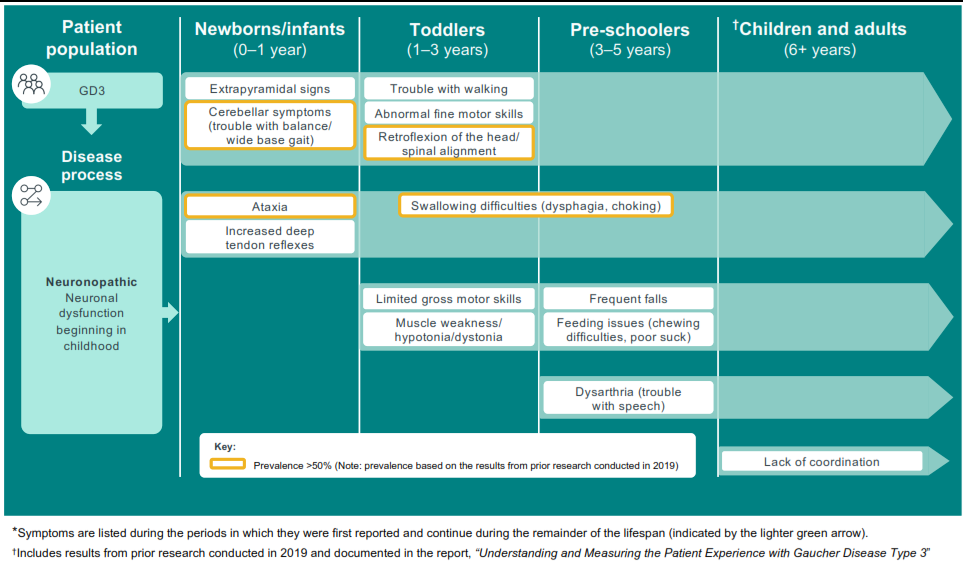


GD3: Gaucher disease type 3

## Fig. S2 Conceptual model for onset of GD3 signs and symptoms associated with cognitive impairment*


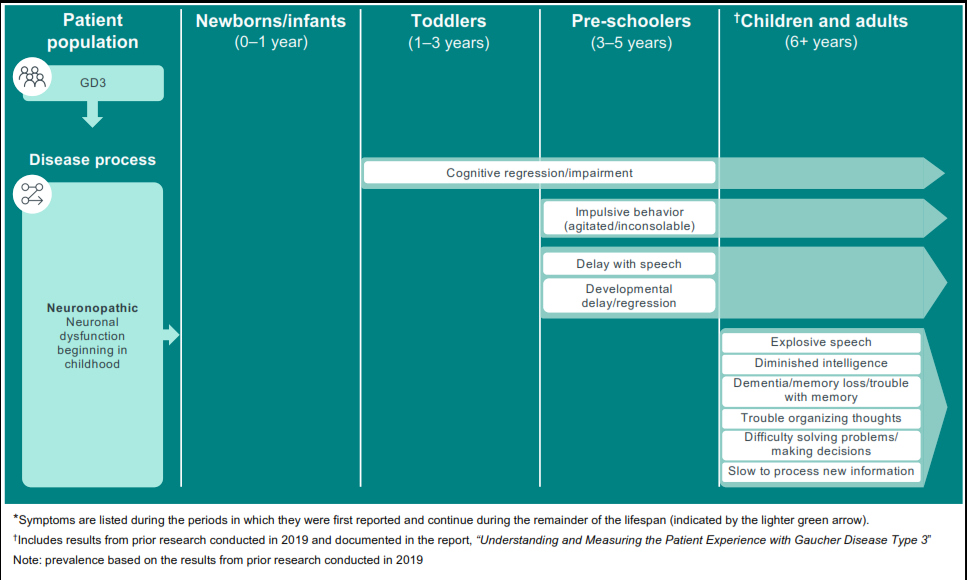

GD3: Gaucher disease type 3
